# The Smart-weed as a Remedy for Mercurial Salivation and Apthous Stomatitis

**Published:** 1848-08

**Authors:** 


*The Smart-weed as a Remedy for Mercurial Salivation and Apthous Stomatitis.* (Communicated in a Letter to the Editor of Am. Jour. Med. Sciences.) By R. WILCOX, M. D., of Elmira, New-York.

I have frequently, during the past year, prescribed a decoction of the leaves of the herb, well known by all in this country, and familiarly denominated, smart-weed, as a remedy in mercurial ptyalism, and also in that form of aphthous stomatitis which occurs during lactation; and the degree of satisfaction I have felt while using it, has only been equalled by my surprise.

The possibility that this plant might possess remedial powers for salivation was suggested to my mind by the fact that farriers employed it with success to the cure of that disease of the salivary glands of the horse called slabbering; and being destitute so far as I then knew of any agent, which, if locally applied, would exert a decided curative effect, I resolved upon its trial the first opportunity. The opportunity soon presented itself. The remedy was used; and I was agreeably surprised when I saw every distressing and unpleasant symptom removed in twenty-four hours.

I have now employed it in about twenty cases of mild mercurial salivation, and have uniformily procured the same prompt and complete relief. I have tried its use in two of the severer forms, attended with numerous and deep ulcerations; these cases were made much more comfortable, but not cured. The benefit I derived from it in salivation induced me to make trial of it in the follicular stomatitis of nurses, which appears to be endemical to the valley of the Chemung River. (At least it is of much more frequent occurrence here, than at any location with which I am acquainted.) Within the last three months, I have prescribed its use in ten cases, and without an exception, they have been speedily and entirely relieved.

I am not prepared to state what can be accomplished with it in those cases of aphthous stomatitis connected with a tubercular state of the constitution, having yet had no such case; neither have I used it where the ulcerations were deep; in such cases, other remedies will doubtless be more effective. I suggest the remedy to my medical brethren for further trial, and if it shall prove in their hands to be worthy the commendation I have given it, I shall ever feel myself abundantly rewarded. The manner in which I have used it, is to take about an ounce of the dried leaves and tops; water, one pint; boil twenty minutes and strain. The mouth is washed with it every hour through the day.

ELMIRA, N. Y., May 8th, 1848.

